# Real-time calcium uptake monitoring of a single renal cancer cell based on an all-solid-state potentiometric microsensor

**DOI:** 10.3389/fbioe.2023.1159498

**Published:** 2023-03-30

**Authors:** Jiali Zhai, Wenting Wang, Shuang Wu, Tianxi Yu, Chongjun Xiang, Yue Li, Chunhua Lin, Guangtao Zhao

**Affiliations:** ^1^ School of Rehabilitation Medicine of Binzhou Medical University, Yantai, China; ^2^ Central Laboratory, The Affiliated Yantai Yuhuangding Hospital of Qingdao University, Yantai, China; ^3^ Department of Urology, The Affiliated Yantai Yuhuangding Hospital of Qingdao University, Yantai, China; ^4^ School of Basic Medicine, Binzhou Medical University, Yantai, China

**Keywords:** single-cell analysis, calcium, ion-selective microelectrodes, tumor microenvironment, renal cell carcinomas

## Abstract

**Introduction:** In addition to many cellular processes, Ca^2+^ is also involved in tumor initiation, progression, angiogenesis, and metastasis. The development of new tools for single-cell Ca^2+^ measurement could open a new avenue for cancer therapy.

**Methods:** The all-solid-state calcium ion-selective microelectrode (Ca^2+^-ISμE) based on carbon fiber modified with PEDOT (PSS) as solid-contact was developed in this work, and the characteristics of the Ca^2+^-ISμE have also been investigated.

**Results:** The Ca^2+^-ISμE exhibits a stable Nernstian response in CaCl_2_ solutions in the active range of 1.0 × 10^−8^ - 3.1 × 10^−3^ M with a low detection limit of 8.9 × 10^−9^ M. The Ca^2+^-ISμE can be connected to a patch clamp to fabricate a single-cell analysis platform for *in vivo* calcium monitoring of a single renal carcinoma cell. The calcium signal decreased significantly (8.6 ± 3.2 mV, n = 3) with severe fluctuations of 5.9 ± 1.8 mV when the concentration of K^+^ in the tumor microenvironment is up to 20 mM.

**Discussion:** The results indicate a severe cell response of a single renal carcinoma cell under high K^+^ stimuli. The detection system could also be used for single-cell analysis of other ions by changing different ion-selective membranes with high temporal resolution.

## 1 Introduction

Single-cell analysis is an interdisciplinary front that grows out of many subjects, such as analytical chemistry (Xu et al., 2016), biology (Zhou et al., 2015), and medical science (Ramanathan et al., 2016). Single-cell analysis could realize the real-time dynamic monitoring of the stress response to changing circumstances in a cell, and it could also avoid the coverage of single-cell change in the average data calculated from the cell populations. Therefore, single-cell analysis plays an important role in understanding cell heterogeneity and biological processes, and developing new tools to promote single-cell analysis studies could help illustrate cellular regulation mechanisms (Fan et al., 2019).

Microelectrodes are powerful tools for electrochemical detection principles, which are excellent candidates to elucidate the response of a single cell. Since the emergence of the microelectrodes, they have been rapidly used in physiological studies, such as membrane potentials of muscle fiber (Adrian, 1956) and giant axons (Hodgkin and Katz, 1949). Nowadays, microelectrodes are widely used in environmental monitoring (Luther et al., 1999; Correia Dos Santos et al., 2001), clinical diagnosis (Abdurhman et al., 2015), and so on. The microelectrodes widely used in single-cell analysis include potentiometric microelectrodes (Simon and Oehme, 1975), voltammetric microelectrodes (Huang et al., 2001), and amperometric microelectrodes (Chen et al., 1994; Cox et al., 2013). Ion-selective microelectrodes are an ideal tool for cellular ion measurements, including intracellular and extracellular ions (Messerli et al., 2007; Donini and O'Donnell, 2005).

Changes in cellular ion concentrations are the early stage of cell toxicological effects under environmental stimuli, especially calcium ion, which is crucial to many (patho)physiological processes. As an important second messenger, calcium ion is a key player in various physiological events, such as neurotransmitter release (Zhao et al., 2019a), muscle contraction (Landstrom et al., 2017), and plant stomatal immunity (Thor et al., 2020). Moreover, calcium is also involved in tumor initiation, progression, angiogenesis, and metastasis (Cui et al., 2017; Monteith et al., 2017). Therefore, calcium ion monitoring at the single-cell level could directly reflect the physiological activities of each cell under the stimuli of the tumor microenvironment, which is critically important for the identification of the specific properties of cancers.

As the third most common genitourinary malignancy, renal cell carcinoma (RCC) represents a heterogeneous group of cancers, more interesting malignancies, and a low survival rate (Weiss and Lin, 2006; Bratslavsky and Linehan, 2010; Vuong et al., 2019). Real-time calcium ion permeation monitoring of a single RCC cell could provide theory instruction for the stress response of RCC cells under the change in the tumor microenvironment. However, research focusing on the ion change at the early stage of cellular stress response of RCC at the single-cell level is rather rare.

The gold standard for the investigation of the permeation of certain ions through the ion channels in a single cell is the patch-clamp technique (Xiao et al., 2012). However, even the patch clamp could record the potential signal of a single cell, but it could not identify certain ions due to the lack of specificity (Toczyłowska-Mamińska et al., 2016). The ion-selective membrane of the potentiometric microelectrode has high specificity owing to the ionophore, which enables the identification of the potential signal generated by certain ions from other ions. The calcium potentiometric microelectrode can be used to register the movement of calcium ions across the cytomembrane with the advantages of wide linear range, high specificity, fast response, and easy manipulation (Simon and Oehme, 1975; Asif et al., 2009). Hence, the combination of the calcium potentiometric microelectrode and patch-clamp technique could be a potential platform for the single-cell analysis of calcium ions with high specificity. In this work, an all-solid-state potentiometric microelectrode based on carbon fiber was fabricated and connected to the patch clamp to record the potential signal of a single renal carcinoma cell, which could provide illustrations for the role of calcium ions in the early stage of the cell response under tumor microenvironmental stimuli.

## 2 Materials and methods

### 2.1 Chemicals

High-molecular weight poly (vinyl chloride) (PVC), 2-nitrophenyl octyl ether (*o*-NPOE), tris (hydroxymethyl)-aminomethane (Tris), poly (sodium 4-styrenesulfonate) (NaPSS, molar mass = 70,000 g mol^-1^), monomer 3,4-ethylenedioxythiophene (EDOT, >97%), and N,N-dicyclohexyl-N′,N′-dioctadecyl-diglycolic diamide (ETH 5234) were purchased from Sigma-Aldrich. Lipophilic cation-exchanger sodium tetrakis [3,5-bis(trifluoromethyl) phenyl] borate (NaTFPB) was purchased from Alfa Aesar. All other chemicals used were of analytical reagent grade. Deionized water (18.2 MΩ cm specific resistance) obtained using a Pall Cascada laboratory water system was used throughout the experiment.

### 2.2 Fabrication of the carbon fiber microelectrode

The fabrication of the carbon fiber microelectrode was according to picture b in the graphical abstract. A carbon fiber with a diameter of 7 μm was attached to a copper wire using silver glue filled with graphite (Enson Company, Guangzhou, China) and then dried at 60°C for 4 h. After being dried, the carbon fiber was carefully inserted into a glass capillary tube pulled earlier to form a tip of ca. 20 μm in diameter, and the length of the glass capillary is ca. 2 cm. Then, the copper wire was pulled into another glass capillary tube with a length of 5 cm, and the adjacent ends of the two glass capillary tubes were both fixed with non-conducting epoxy glue. The other end of the glass capillary tube with a length of 5 cm was empty and filled with silver conductive paint (Mechanic Company, Hong Kong, China). Afterward, the microelectrode was connected to the patch clamp (Axon 700B, Molecular Devices, United States), while the silver wire of the patch clamp was put into the capillary glass tube with the microelectrode. The carbon fiber was flame-fuse-sealed at the tip of the capillary, and the protruding carbon fiber can be easily etched to form the carbon fiber microelectrode, which is denoted as CFμE.

### 2.3 The electrodeposition of the PEDOT (PSS) films

The poly (3,4-ethylenedioxythiophene)-poly (sodium 4-styrenesulfonate) (PEDOT (PSS)) films were electrodeposited on the surface of the CFμE by galvanostatic electrochemical polymerization in deaerated 0.1 M EDOT + 0.01 M NaPSS solution to prepare the CFμE/PEDOT (PSS) electrode. A constant current of 5 × 10^−8^ A was applied for 100 s to produce polymerization charges of 5 μC. The polymerization was performed in a conventional three-electrode cell. A Pt wire was used as a counter electrode, an Ag/AgCl/3 M KCl microelectrode was used as the reference electrode, and the CFμE was used as the working electrode (Bobacka, 1999; Vanamo et al., 2016).

### 2.4 Fabrication of the Ca^2+^-ISμE

The Ca^2+^-selective membrane composition was 1.3% (wt) ETH 5234, 0.6% (wt) NaTFPB, 65.3% (wt) *o*-NPOE, and 32.8% (wt) PVC. A measure of 100 mg of the membrane components was dissolved in 0.8 mL of THF. The CFμE/PEDOT (PSS) electrodes were rinsed with deionized water after electrodeposition and allowed to dry in the air for 1 day. Then, each electrode was dipped eight times in the Ca^2+^-selective membrane solution and allowed to dry for 15 min at room temperature after each dip. After being dried, the all-solid-state Ca^2+^-ISμE, denoted as CFμE/PEDOT (PSS)/Ca^2+^-ISE, was conditioned in 10^−4^ M CaCl_2_ solution for 0.5 h before use. For comparison, the bare CFμE was covered with a Ca^2+^-selective membrane solution to prepare CFμE/Ca^2+^-ISE (Sokalski et al., 1999). All measurements of electromotive force (EMF) were carried out at room temperature using a CHI660E electrochemical station (Shanghai Chenhua Apparatus Corporation, China) with an Ag/AgCl/3 M KCl microelectrode as the reference electrode. The EMF values were corrected for the liquid-junction potentials using the Henderson equation. The ion coefficient activities were calculated using the Debye–Hückel equation (Kamata et al., 1988). For selectivity measurements of the Ca^2+^ ion-selective membrane toward the interfering ions in the tumor microenvironment, the glassy carbon electrodes (GCEs, 3 mm in diameter) modified with PEDOT (PSS) were used to prepare the GC/PEDOT (PSS)/Ca^2+^-ISEs, as described in the previous work (Zhao et al., 2019b).

### 2.5 Apparatus and measurements

The renal cell carcinoma tissue samples were collected from in-patients in the Affiliated Yantai Yuhuangding Hospital of Qingdao University for calcium measurement through the single-cell platform composed of the CFμE/PEDOT (PSS)/Ca^2+^-ISE and patch clamp. The tissue samples were cut into slices with a thickness of 300 μm. Then, all the renal cancer slices were incubated in RPMI 1640 media before measurement. Hank’s solution (NaCl 8.01 g/L; KCl 0.4 g/L; CaCl_2_ 0.14 g/L; NaHCO_3_ 0.35 g/L; KH_2_PO_4_ 0.06 g/L; and glucose 0.34 g/L) served as the electrolyte in the microenvironment around the cell. A piece of renal cell carcinoma slice was washed with Hank’s solution three times before being placed into the cell of the patch clamp filled with 2 mL of Hank’s solution, and the tip of the CFμE/PEDOT (PSS)/Ca^2+^-ISE was positioned onto the cytomembrane of one single renal carcinoma cell using a micromanipulator. The calcium potential signal of the single renal carcinoma cell was first recorded for ca. 10 min and then for another 30 min twice, respectively, after each addition of an appropriate volume of high KCl stock solution to obtain the final concentration of 10 mM and 20 mM.

## 3 Results and discussion

### 3.1 Modification of the PEDOT (PSS) film

**FIGURE 1:**
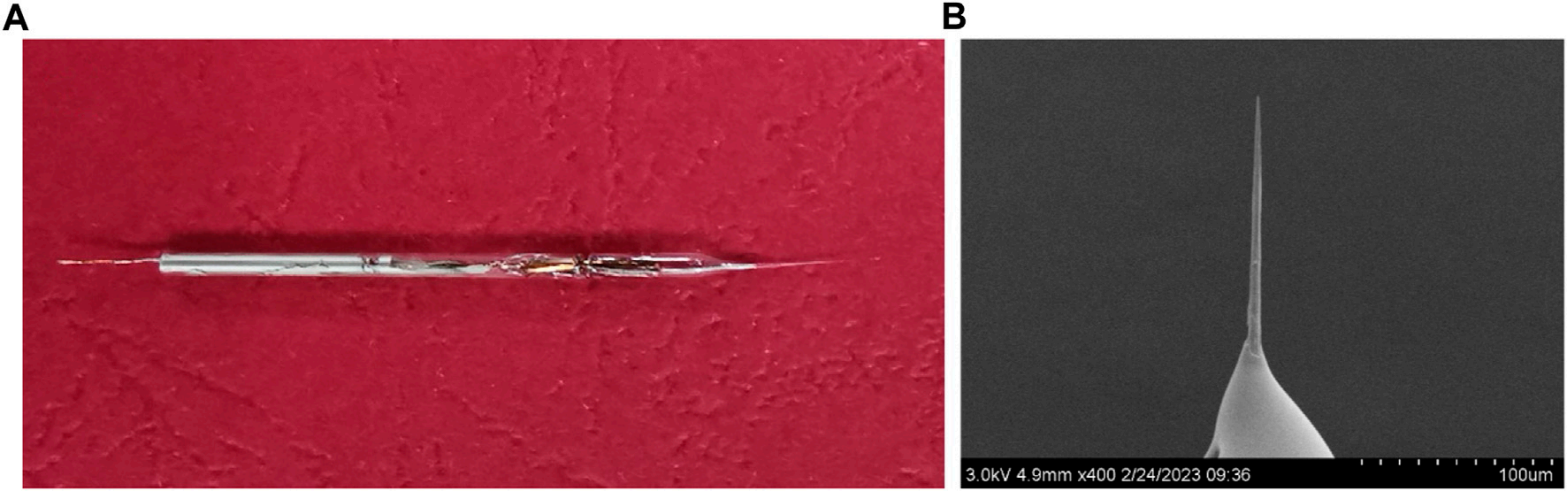
**(A)** Photograph of the CFμE and **(B)** scanning electron microscopy picture of the CFμE tip.

**FIGURE 2:**
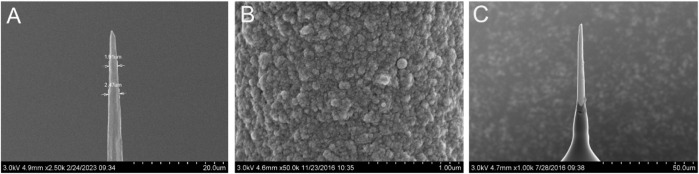
SEM pictures of the CFμEs. **(A)** CFμE tip, **(B)** PEDOT (PSS) film, and **(C)** CFμE tip covered with the polymeric membrane.

Figure 1A shows the general view of the CFμE, and the tip of the CFμE is shown in Figure 1B. The figure shows a smooth carbon fiber tightly sealed in the capillary of the electrode. The diameter of the electrode tip is 1.91 μm, as shown in Figure 2A. The voltammetry characteristics of the CFμEs are shown in Supplementary Figure S1, and only CFμEs that displayed sigmoid-shaped voltammograms were selected for further analysis. The PEDOT (PSS) film was electrodeposited onto the surface of the CFμEs as an ion-to-electron transducer owing to its good electrochemical stability in its oxidized (doped) state (Zhou et al., 2010). The PEDOT (PSS) film surface consisted of well-separated globules which are similar to the globular morphology reported in the literature (Figure 2B) (Zhou et al., 2010). The CFμE/PEDOT (PSS) was covered with a Ca^2+^-selective membrane, and the smooth surface of the Ca^2+^-ISμE is shown in Figure 2C.

### 3.2 CV and EIS

**FIGURE 3:**
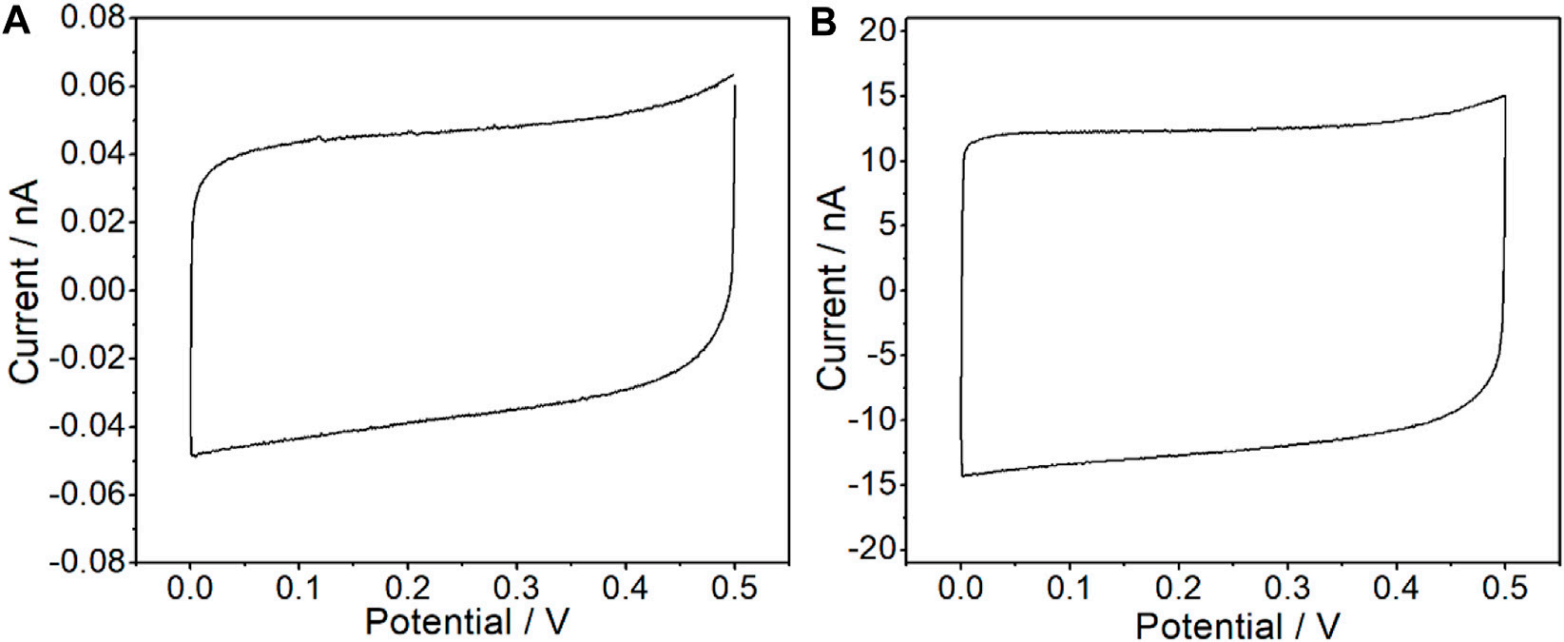
Cyclic voltammograms recorded in 0.1 M KCl for the bare CFμEs **(A)** and the CFμEs modified with PEDOT (PSS) composite **(B)**. The scan rate is 50 mV/s.

Cyclic voltammetry (CV) and electrochemical impedance spectroscopy (EIS) measurements were carried out in 0.1 M KCl solution through a CHI660E electrochemical station to investigate the electrochemical characteristics of the CFμEs and CFμE/PEDOT (PSS). As shown in Figure 3A, a high reversibility for the doping process of the PEDOT (PSS) film could be revealed from a capacitive process with a symmetrical near-rectangular shape of the CV from 0 to 0.5 V (Ocaña et al., 2018). The capacitive current of the CFμEs was only at the 4 × 10^−11^ A level because of the high resistance, while the CFμE/PEDOT (PSS) was 200-fold higher than the bare microelectrode (Figure 3B). This phenomenon indicates that the redox capacitance of the electrode could be enhanced due to the presence of the PEDOT (PSS) film, which is in accordance with the conventional electrode (Yin et al., 2012). In order to calculate the effective surface area of the CFμE, CV was carried out in 5 mM [Fe (CN)_6_]^3-/4-^ solution in the background of 0.1 M KCl (Supplementary Figure S2). According to the Randles–Sevcik equation: ip = 2.69 × 10^5^ n^3/2^ A D^1/2^ V^1/2^ C_o_, where ip is the peak current (A), n is the number of electrons, A is the electrode area, D is the diffusion coefficient 6.7 × 10^−6^ (cm^2^ S^−1^), V is the scan rate (V s^−1^), and C_o_ is the concentration (mol cm^−3^) (Rizwan et al., 2018). The effective surface area of the CFμE was calculated to be 8.9 × 10^−11^ cm^2^, and the effective surface area of the electrode increased to 15.5 × 10^−11^ cm^2^ after the modification of the PEDOT (PSS) film.

**FIGURE 4:**
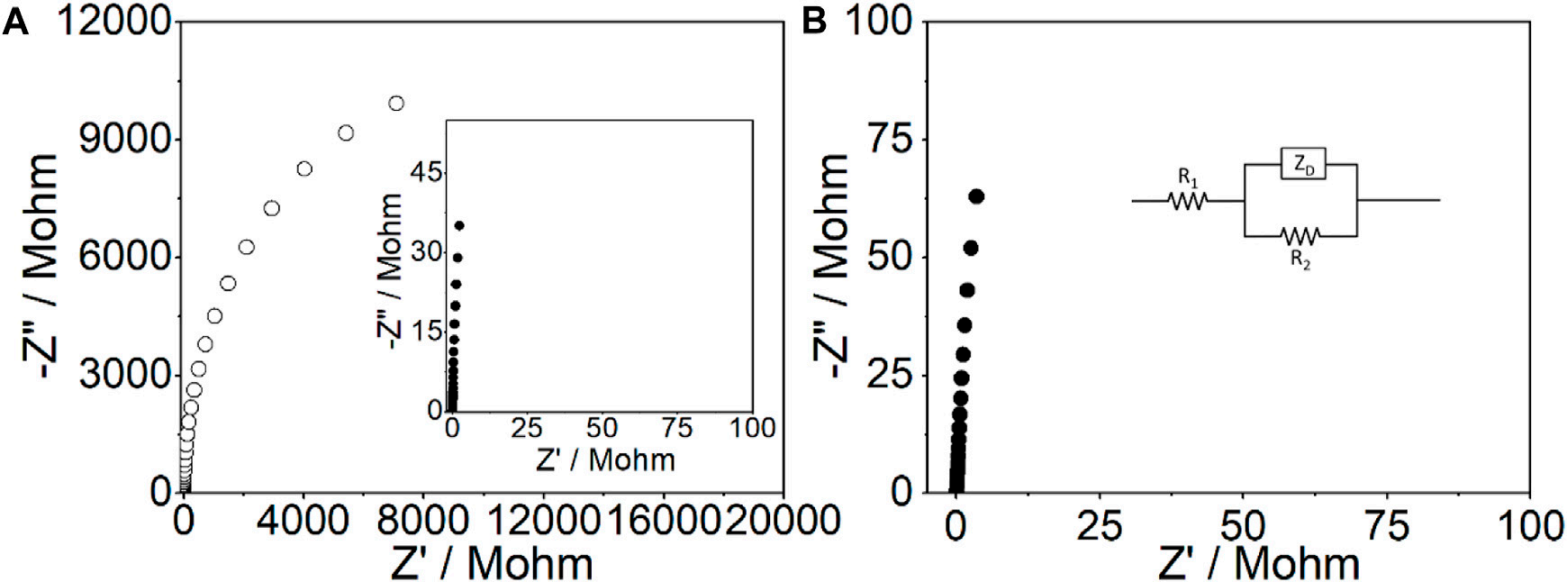
Impedance spectra for the bare CFμEs (○) and the CFμEs modified with the PEDOT (PSS) film (●) **(A)** and the Ca^2+^-ISμE **(B)** in 0.1 M KCl solution at the open-circuit potential. Frequency range: 0.01 Hz to 10 kHz; excitation amplitude: 100 mV. In the inset of Figure 4B, R_1_ is the solution resistance, Z_D_ is the finite-length diffusion impedance, and R_2_ is the parallel resistance.

The impedance spectra of the CFμE and CFμE/PEDOT (PSS) both indicate fast charge transfers at the interfaces for the near-90° capacitive lines with the absence of the high-frequency semicircle (Figure 4A). According to the equation, C = −1/(2πfZ″), where f is the lowest frequency used to record the spectra (0.01 Hz) and Z″ is the impedance at this frequency (Zhou et al., 2010; Ocaña et al., 2018). The redox capacitance of the CFμE and CFμE/PEDOT (PSS) was calculated to be 1.6 ± 0.2 nF and 453.4 ± 9.7 nF, respectively. This phenomenon agrees well with the results obtained by CV, where CFμE/PEDOT (PSS) shows much higher redox capacitance than the bare CFμE. Therefore, serving as the ion-to-electron transducer of the all-solid-state Ca^2+^-ISμE, the PEDOT (PSS) film has sufficiently high bulk (redox) capacitance. The impedance spectra of the CFμE/PEDOT (PSS)/Ca^2+^-ISE were also recorded in 0.1 M KCl solution (Figure 4B), and the bulk capacitance of the CFμE/PEDOT (PSS)/Ca^2+^-ISE was calculated to be 256.3 ± 28.2 nF when fitting the equivalent circuit (average error, χ^2^ = 0.09), which is lower than that of the CFμE/PEDOT (PSS) (458.7 ± 32.9 nF), and this phenomenon may be due to the coating of the calcium ion-selective membrane.

### 3.3 Characteristics of the Ca^2+^-ISμE

**FIGURE 5:**
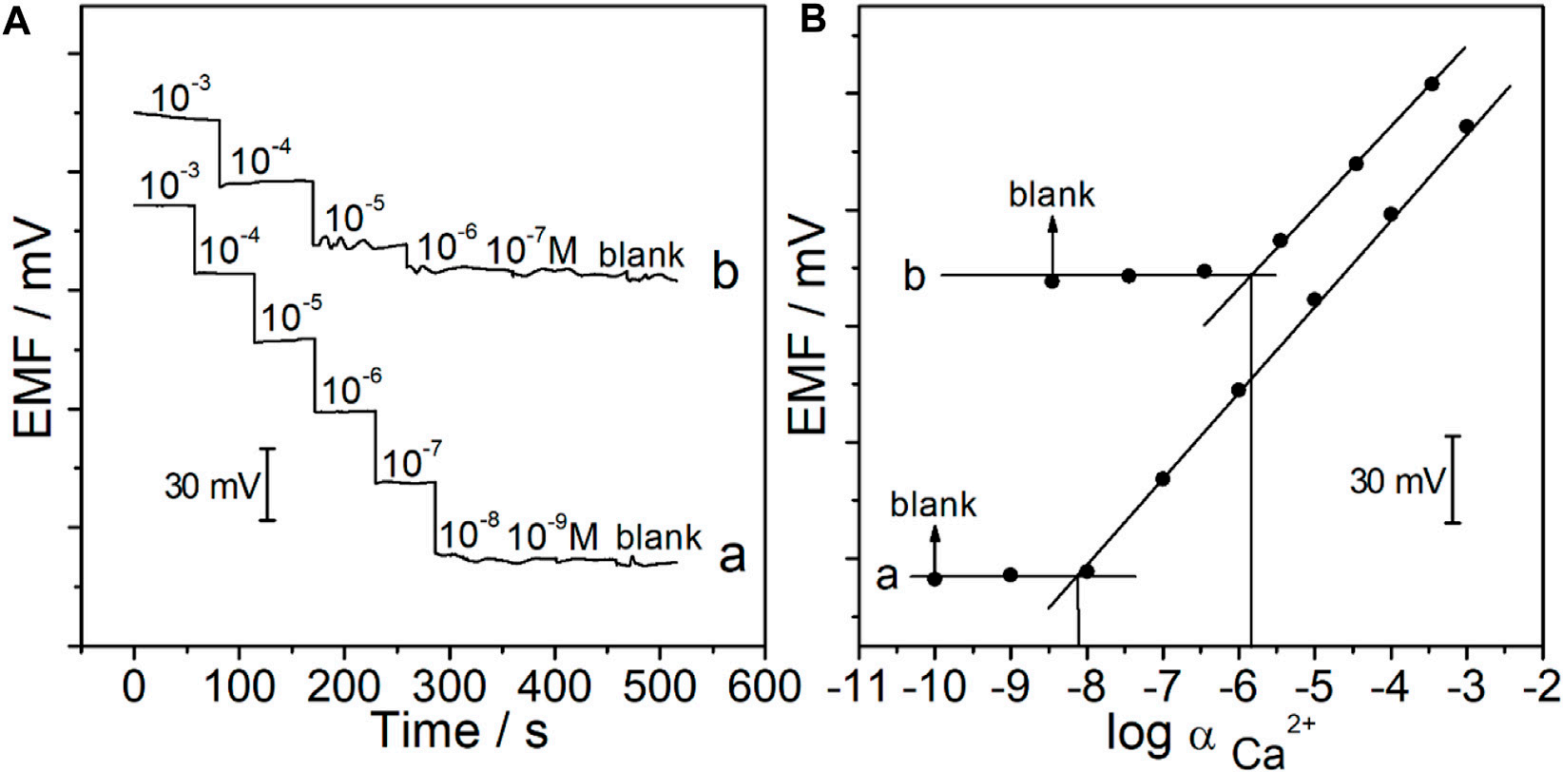
**(A)** Potential time trace of the CFμEs/PEDOT (PSS)/Ca^2+^-ISE in CaCl_2_ solutions at different concentrations in the absence **(A)** and presence **(B)** of Hank’s solution. **(B)** Calibration curve of the CFμEs/PEDOT (PSS)/Ca^2+^-ISE in the absence **(A)** and presence **(B)** of Hank’s solution.

The potentiometric response of the CFμE/PEDOT (PSS)/Ca^2+^-ISE was measured in the active range of 1.0 × 10^−9^–3.1 × 10^−3^ M CaCl_2_. As shown in Figure 5A, the CFμE/PEDOT (PSS)/Ca^2+^-ISE exhibits a stable Nernstian response, and the linear range was from 1.0 × 10^−8^ to 3.1 × 10^−3^ M with a slope of 29.3 ± 1.4 mV/decade (*R* ^2^ = 0.9995). The detection limit was 8.9 × 10^−9^ M which was calculated from the intersection of the two lines (Figure 5B). The reproducibility of the proposed electrodes was investigated by alternatively measuring 10^−4^ M and 10^−3^ M CaCl_2_ (Supplementary Figure S3). The standard deviations of the potential values were ±0.4 mV for 10^−4^ M CaCl_2_ and ±0.9 mV for 10^−3^ M CaCl_2_. Herein, the prepared CFμE/PEDOT (PSS)/Ca^2+^-ISE has good reproducibility (Rubinova et al., 2007).

Current-reversal chronopotentiometry was carried out to investigate the short-term potential stabilities of CFμE/PEDOT (PSS)/Ca^2+^-ISE and CFμE/Ca^2+^-ISE (Supplementary Figure S4). The potential drift of the CFμE/PEDOT (PSS)/Ca^2+^-ISE was calculated to be 10.8 ± 4.4 µVs^-1^, which is much lower than that of the CFμE/Ca^2+^-ISE (160.8 ± 36.3 µVs^-1^) under the applied currents of ±0.01 nA. Therefore, the potential stability of the microelectrodes was dramatically improved by the PEDOT (PSS) film (Yin et al., 2012). As shown in Supplementary Figure S5, the spontaneous formation of a water layer between the membrane and the surface of a carbon fiber microelectrode could cause the potential instabilities of the CFμE/Ca^2+^-ISE (Bakker and Pretsch, 2005). However, as for the CFμE/PEDOT (PSS)/Ca^2+^-ISE, there was no potential shift when the measured solutions were changed, which indicates that the water layer could be efficiently reduced by the electrodeposition of the PEDOT (PSS) film. After the condition, the CFμE/PEDOT (PSS)/Ca^2+^-ISE could maintain the Nernstian response for at least 48 h while being stored at 4°C.

The effect of tumor microenvironment pH on the potential response of Ca^2+^-ISμE was investigated by measuring 10^–3^ M CaCl_2_ in the presence of Hank’s solution over a wide pH range (Supplementary Figure S6). As shown in Supplementary Figure S6, the EMF value of Ca^2+^-ISμE remained stable at pH values ranging from 5.0 to 8.0, and the potential values at high pH 9.0 showed a little deviation, which may be due to the OH^−^ ions. The influence of the temperature on the potential response of Ca^2+^-ISμE was also investigated by measuring 10^–3^ M CaCl_2_ in the presence of Hank’s solution over a wide range of 10°C–40°C (Supplementary Figure S7), and the result shows that the Ca^2+^-ISμE could maintain a stable EMF value at a wide range.

### 3.4 *In vivo* monitoring of Ca^2+^ change in a single cell

**FIGURE 6:**
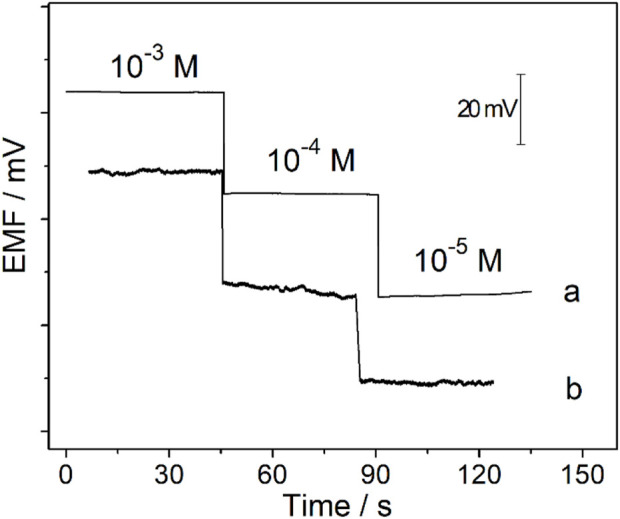
Potential responses of the Ca^2+^-ISμE recorded in CaCl_2_ solutions ranging from 1 × 10^−5^ to 1 × 10^−3^ M through the CHI660E electrochemical station (a) and the patch clamp (b).

**FIGURE 7:**
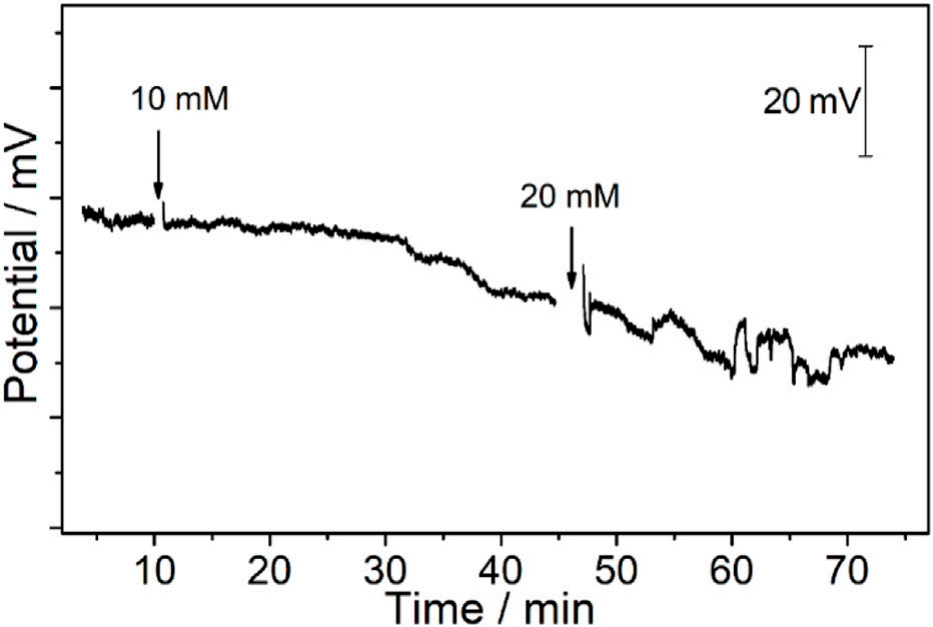
Potentiometric time trace of the CFμEs/PEDOT (PSS)/Ca^2+^-ISE toward Ca^2+^ after the electrode is placed on the cytomembrane of the renal carcinoma cell. The arrow points to the time point of adding different concentrations of K^+^ to obtain final concentrations of 10 mM and 20 mM.

After being conditioned in 10^−5^ M CaCl_2_ solution for 0.5 h, the Ca^2+^-ISμE was connected to the patch clamp, and the potential response of the Ca^2+^-ISμE was detected in CaCl_2_ solutions through the patch clamp. The results show that the Ca^2+^-ISμE could also show a Nernstian response in CaCl_2_ solutions in the range of 10^−5^–10^−3^ M (Figure 6a, Figure 7), which is in good accordance with the potential response obtained by the CHI660E electrochemical workstation (Figure 6a). Therefore, the Ca^2+^-ISμE was well connected to the patch clamp and could be used for recording the calcium potential signal of a single cell. Moreover, the time interval of the potential signal recorded by the patch clamp was only 0.1 ms. Therefore, the prepared all-solid-state Ca^2+^-ISμE could record the acute calcium ion signal of a single renal carcinoma cell with high temporal resolution.

The electrolyte of the tumor microenvironment is vitally important for the cell as it must stay in a certain range to maintain the cell osmotic pressure, which has a direct relationship with cell behaviors and even cell survival. Hence, the imbalance of the electrolyte of the tumor microenvironment has a direct relationship with the cellular processes, especially high K^+^. As the most abundant intracellular cation, potassium plays a crucial role in many cellular functions, including neurotransmitter release, blood pressure, maintaining fluid and electrolyte balance, and kidney diseases (Raebel, 2012; Ren et al., 2017). Moreover, K^+^ channels could also regulate human T-cell activation and proliferation by triggering downstream Ca^2+^-dependent pathways (Petsakou and Perrimon, 2022). Herein, the monitoring of calcium ions of a single cell under high K^+^ is critically important for cancer therapies, which is helpful for the illustration of the role of calcium signal in single renal carcinoma cellular response under the microenvironment stimuli.

The RCC tissue slices were placed in Hank’s solution which served as the electrolyte in the tumor microenvironment. For the measurement of calcium ions, the selectivity coefficients of the Ca^2+^-ISM toward various interfering ions in the tumor microenvironment were examined by using the separate solution method (Bakker et al., 2000). As shown in Supplementary Figure S8, the results obtained are consistent with those of the Ca^2+^-ISE in the literature, including Na^+^, K^+^, Mg^2+^, H^+^, and Zn^2+^ (Sokalski et al., 1999). The selectivity coefficient toward sodium ions which is the most abundant ion in the tumor microenvironment was log $K_{Ca,Na}^{pot}$ = −5.2, and a theoretical detection limit for calcium ions in the presence of 0.14 M Na^+^ was calculated to be 2.4 × 10^−6^ (Ceresa et al., 2001). The CFμE/PEDOT (PSS)/Ca^2+^-ISE showed a Nernstian response toward calcium ions in Hank’s solution with the absence of Ca^2+^ in the linear range of 3.6 × 10^−6^–3.6 × 10^−4^ M with the slope of 26.9 ± 2.1 mV/decade (*R* ^2^ = 0.9997), and the detection limit was 1.6 × 10^−6^ M (Figure 5A, B), which is close to the theoretical detection limit. Herein, the CFμE/PEDOT (PSS)/Ca^2+^-ISE has good sensitivity and selectivity for the detection of calcium ions even in an environment like Hank’s solution containing other ions in high concentration.

The RCC tissue was cut into slices that could maintain its original state in the body. Meanwhile, the thickness of the RCC tissue was only 300 μm, which could expose a single renal carcinoma cell to realize the *in vivo* calcium analysis at a single-cell level. Moreover, the tip of the Ca^2+^-ISμE on a single renal carcinoma cell is shown in Supplementary Figure S9, which indicates that the calcium signal was recorded from one single cell. As shown in Figure 5, the calcium signal of one single renal carcinoma cell almost remained at a stable state with small fluctuations of less than 2 mV before the addition of high K^+^, which demonstrates that the renal carcinoma cell almost remains at a resting state without any stimuli. A decrease in the calcium potential signal (ca. 6.0 ± 3.6 mV) could be observed when the extracellular concentration of K^+^ was up to 10 mM. The concentration of calcium in Hank’s solution was 1.3 mM, and according to the slope of the electrodes (26.9 ± 2.1 mV/decade), the concentration of calcium around the cell decreased by 0.22 ± 0.13 mM, while the concentration of K^+^ above a critical threshold (20 mM) could lead to not only a potential decrease of 8.6 ± 3.2 mV (*n* = 3) but also fluctuations of 5.9 ± 1.8 mV, which indicates a decreased calcium concentration of 0.32 ± 0.12 mM and fluctuations of 0.22 ± 0.07 mM. This phenomenon indicates a severe response of the renal carcinoma cell with high amounts of calcium ion permeation through the cytomembrane under high K^+^ stimuli.

The Ca^2+^-ISμE could also maintain a Nernstian response after single-cell measurement within 6 h. However, more research on the downstream signaling molecules or cellular behaviors caused by severe fluctuations of the calcium ions would be carried out in future work to provide a comprehensive illustration for cancer therapies. Moreover, the proposed single-cell analysis system composed of the all-solid-state potentiometric microelectrode and patch clamp could be extended for single-cell analysis of other ions, such as K^+^, Na^+^, H^+^, Zn^2+^, and Mg^2+^.

## 4 Conclusion

The proposed all-solid-state Ca^2+^-ISμE based on carbon fiber modified with PEDOT (PSS) shows good reproducibility, good potential stability, and high sensitivity. The Ca^2+^-ISμE could be well connected to the patch clamp and shows a Nernstian response in CaCl_2_ solutions with high temporal resolution. The single-cell analysis platform composed of the Ca^2+^-ISμE and patch clamp could accurately record the calcium uptake of a single renal carcinoma cell on the cytomembrane, and the renal carcinoma cell represents a severe response under the extracellular concentration of K^+^ up to 20 mM in the tumor microenvironment. The single-cell analysis platform is available for single-cell monitoring of other ions by changing different ion-selective membranes accordingly.

## Data Availability

The original contributions presented in the study are included in the article/Supplementary Material; further inquiries can be directed to the corresponding authors.
