# Supplementary material for: Real-time calcium uptake monitoring of a single renal cancer cell based on an all-solid-state potentiometric microsensor
Source: Front Bioeng Biotechnol. 2023 Mar 30;11:1159498. doi: 10.3389/fbioe.2023.1159498 (PMC10098084; doi:10.3389/fbioe.2023.1159498)
Supplement: Supplementary file 1 [file DataSheet1.pdf]

# Real time calcium uptake monitoring of single renal cancer cell based on an all-solid-state potentiometric microsensor

Jiali Zhai[1,†], Wenting Wang[2,†], Shuang Wu[3], Tianxi Yu[3], Chongjun Xiang[3], Yue Li[3], Chunhua Lin[3,*], and Guangtao Zhao[4,**]

[1] *School of Rehabilitation medicine of Binzhou Medical University, Yantai 264003 (China)*

[2] *Central Laboratory, the Affiliated Yantai Yuhuangding Hospital of Qingdao University, Yantai 264000 (China)*

[3] *Department of Urology, the Affiliated Yantai Yuhuangding Hospital of Qingdao University, Yantai 264000 (China)*

[4] *School of Basic Medicine, Binzhou Medical University, Yantai 264003 (China)*

* Corresponding author.

** Corresponding author.

E-mail address: Chunhua.lin@qdu.edu.cn(C. Lin), gtzhao@bzmc.edu.cn(G. Zhao).

[†] These authors have contributed equally to this work.

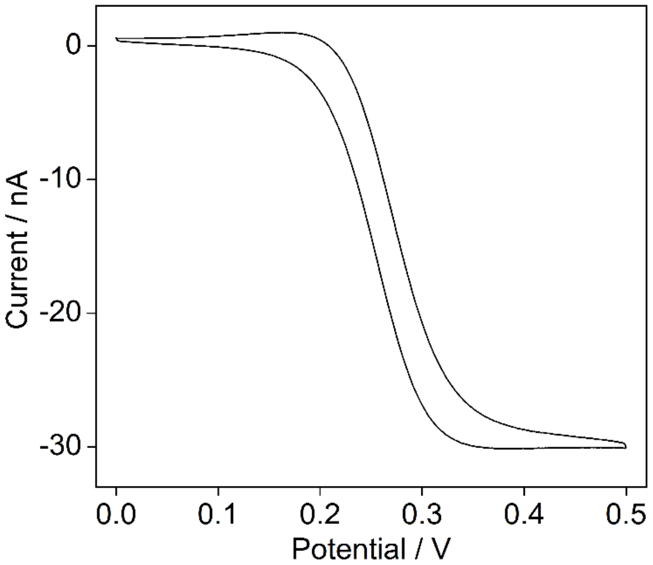


Fig. S1. Cyclic voltammograms for the recorded in 4 mM $K_4[Fe(CN)_6]$ solution (1 M KCl, pH 7.0). The scan rate was 50 mV $s^{-1}$.

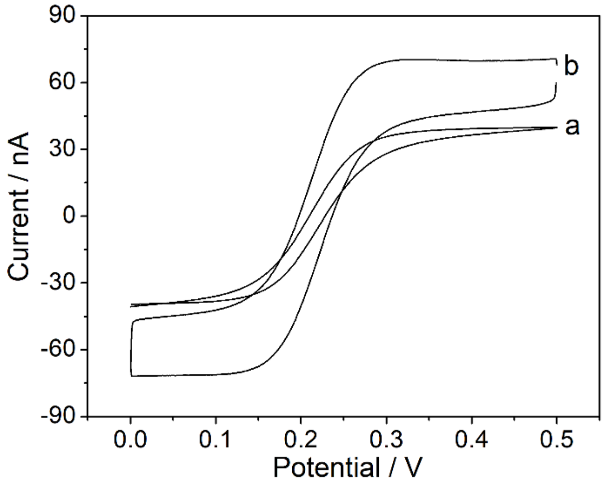


Fig. S2. Cyclic voltammograms recorded in 5 mM mM $[Fe\ (CN)_6]^{3-/4-}$ containing 0.1 M KCl for the CFμEs (a) and CFμEs/PEDOT(PSS) (b). The scan rate is 50 mV/s.

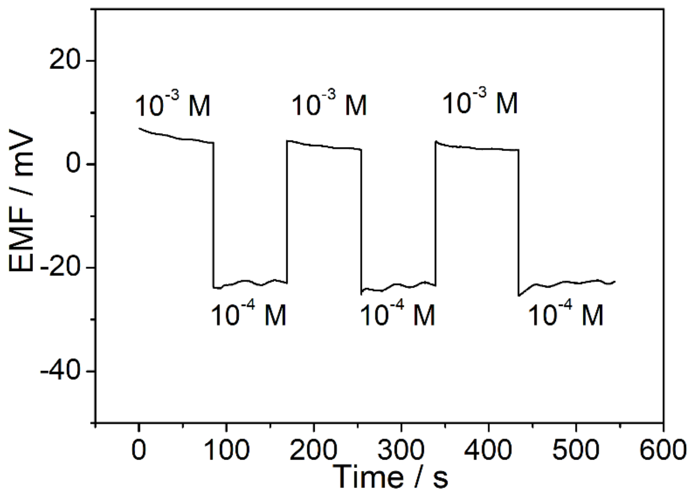


Fig. S3. Potential reproducibility of the CFμEs/PEDOT(PSS)/$Ca^{2+}$-ISE evaluated by alternatively measuring $10^{-4}$ and $10^{-3}$ $CaCl_2$ solutions ($n$=3).

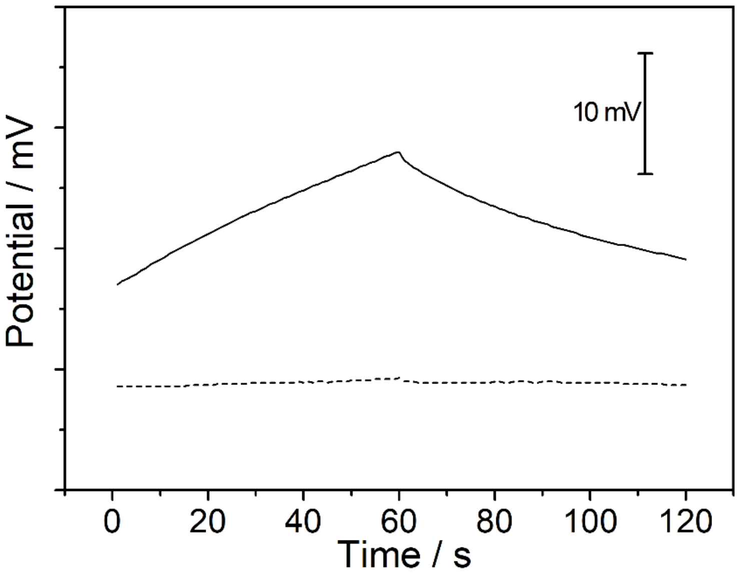


Fig.S4. Chronopotentiograms for the CFμEs/PEDOT(PSS)/$Ca^{2+}$-ISE (*dotted line*) and CFμEs/$Ca^{2+}$-ISE (*solid line*) recorded in 1.0 × $10^{-5}$ M $CaCl_2$. The applied currents were + 0.01 nA for 60 s and - 0.01 nA for another 60 s.

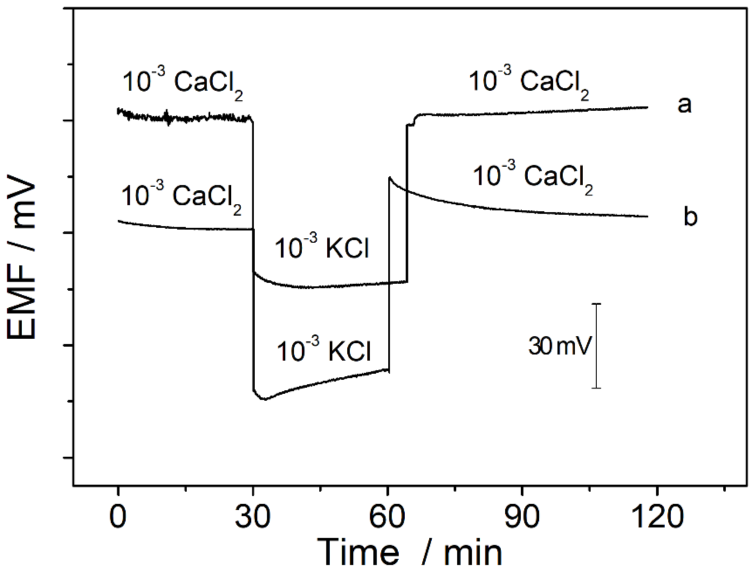


Fig. S5. Water layer tests for the CFμEs/PEDOT(PSS)/$Ca^{2+}$-ISE (a) and CFμEs/$Ca^{2+}$-ISE (b).

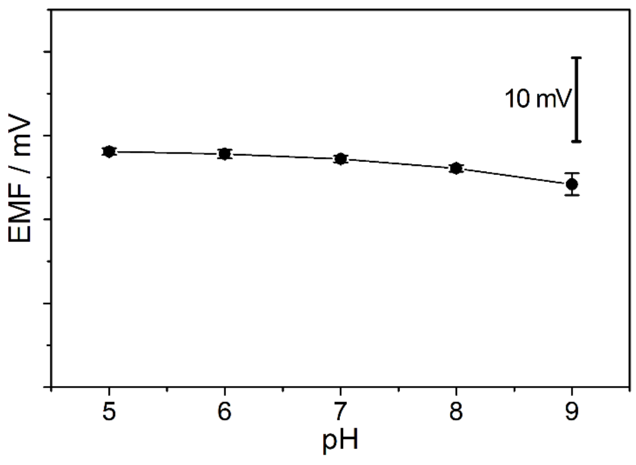


Fig. S6. Potentiometric pH responses of $Ca^{2+}$-ISE in 1.0 × $10^{-3}$ M $CaCl_2$ in the presence of Hank's solution at pHs ranging from 5 to 9.

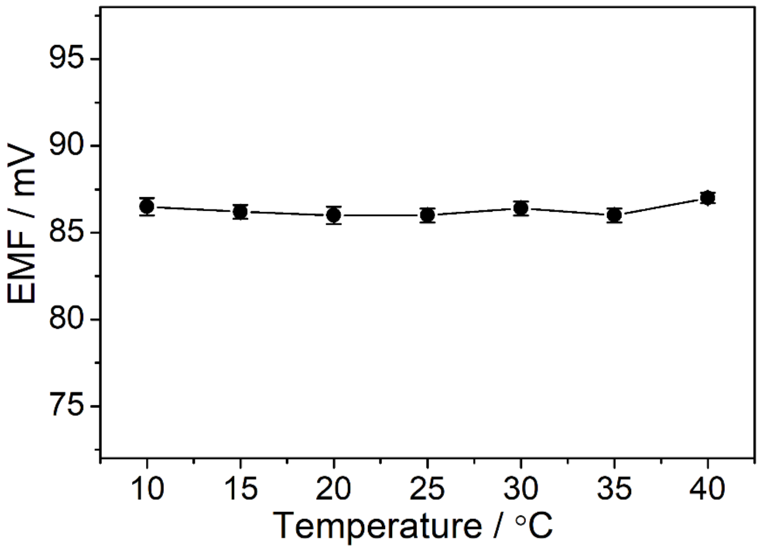


Fig. S7. Potentiometric temperature responses of $Ca^{2+}$-ISE in $1.0 \times 10^{-3}$ M $CaCl_2$ in the presence of Hank's solution at temperature ranging from 10 ˚C to 40˚C.

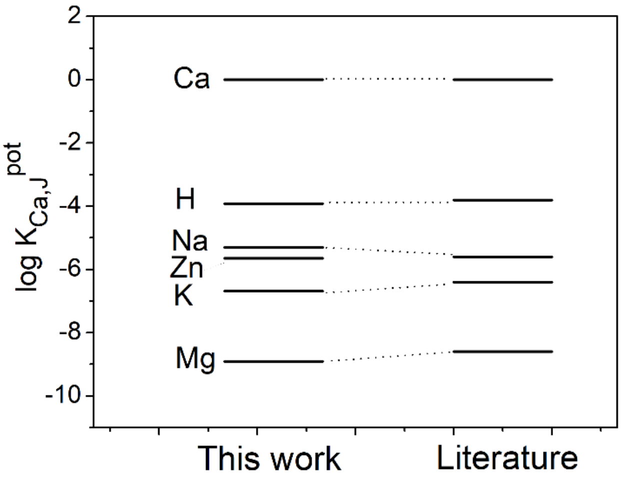


Fig. S8. Comparison of the selectivity coefficients for the present $Ca^{2+}$-selective membrane obtained with the separate solution method [1] and reported in the literature [2].

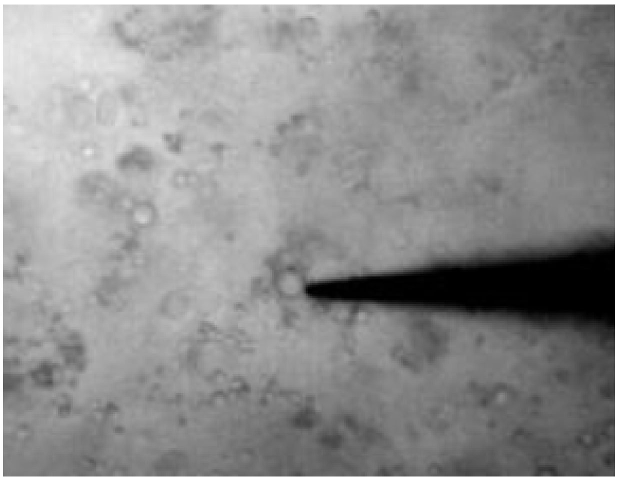

Fig. S9. Micrograph of single renal cancer cell calcium monitoring through the prepared $Ca^{2+}$-ISμE.

**References:**

[1] E. Bakker, E. Pretsch, P. Bühlmann, Selectivity of potentiometric ion sensors, Anal. Chem. 6 (2000) 1127-1133.

[2] T. Sokalski, A. Ceresa, M. Fibbioli, T. Zwickl, E. Bakker, E. Pretsch, Lowering the detection limit of solvent polymeric ion-selective membrane electrodes. 2. influence of composition of sample and internal electrolyte solution, Anal. Chem. 6 (1999) 1210-1214.
